# Feline APOBEC3s, Barriers to Cross-Species Transmission of FIV?

**DOI:** 10.3390/v10040186

**Published:** 2018-04-10

**Authors:** Zeli Zhang, Qinyong Gu, Daniela Marino, Kyeong-Lim Lee, Il-Keun Kong, Dieter Häussinger, Carsten Münk

**Affiliations:** 1 Clinic for Gastroenterology, Hepatology, and Infectiology, Medical Faculty, Heinrich-Heine-University Düsseldorf, 40225 Düsseldorf, Germany; Zeli.Zhang@med.uni-duesseldorf.de (Z.Z.); Qinyong.Gu@med.uni-duesseldorf.de (Q.G.); danielamarino@gmx.de (D.M.); Haeussinger@med.uni-duesseldorf.de (D.H.); 2 Division of Applied Life Science (BK21Plus), Graduate School of Gyeongsang National University, Jinju 52828, Korea; 0920-0728@hanmail.net (K.-L.L.); ikong7900@gmail.com (I.-K.K.)

**Keywords:** FIV, restriction factors, feline APOBEC3, cross-species transmission

## Abstract

The replication of lentiviruses highly depends on host cellular factors, which defines their species-specific tropism. Cellular restriction factors that can inhibit lentiviral replication were recently identified. *Feline immunodeficiency virus* (FIV) was found to be sensitive to several feline cellular restriction factors, such as apolipoprotein B mRNA-editing enzyme, catalytic polypeptide-like 3 (APOBEC3) and tetherin, but FIV evolved to counteract them. Here, we describe the molecular mechanisms by which feline APOBEC3 restriction factors inhibit FIV replication and discuss the molecular interaction of APOBEC3 proteins with the viral antagonizing protein Vif. We speculate that feline APOBEC3 proteins could explain some of the observed FIV cross-species transmissions described in wild Felids.

## 1. Introduction

**Figure 1:**
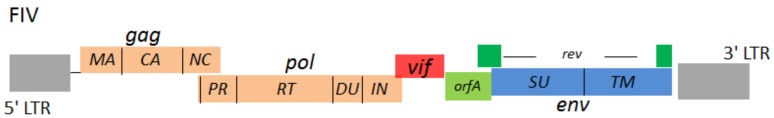
Cartoon representation of *Feline immunodeficiency virus* (FIV) genome structure. Two long terminal repeats (LTRs) locate at 5’ and 3’ termini. The structural genes of FIV are *gag*, *pol*, and *env*. The regulatory genes of FIV are *vif*, *orfA* and *rev*. The *gag* encodes virus structural proteins: matrix (MA), capsid (CA), nucleocapsid (NC). The *pol* encodes virus enzymes: protease (PR), reverse transcriptase (RT), integrase (IN) and dUTPase (DU). The *env* encodes virus envelope proteins: surface (SU) glycoprotein and transmembrane (TM) protein.

*Feline immunodeficiency virus* (FIV) is a lentivirus that attacks the cat immune system and was first isolated in 1986 from a feline leukemia virus (FeLV)-negative cat at the University of California [1]. Worldwide, around 2.5–4.4% of cats are infected with FIV [2]. FIV from the domestic cat is classified into five subtypes (subtypes A–E) [3], mainly based on their envelope gene polymorphisms. FIV is able to infect several kinds of feline immune cells, such as T lymphocytes, monocytes/macrophages, B lymphocytes, and dendritic cells. Unlike human immunodeficiency virus type 1 (HIV-1) that uses CD4 and CXCR4/CCR5 receptors for viral entry, FIV hijacks cellular CD134 as a receptor and CXCR4 as a co-receptor [4,5,6]. CD134 (also known as OX40) is a member of the tumor necrosis factor receptor superfamily and functions as a secondary co-stimulator in regulating T cell response triggered by the T cell receptor (TCR) [7]. CD134 is not constitutively expressed on resting T cells, but is expressed after activation. The genome of FIV contains *gag*, *pol*, *env*, *vif*, *orfA*, and *rev* genes that encode Gag, Pol, Envelope structural and enzymatic polyproteins, the accessory proteins Vif and OrfA, and transactivator protein Rev. (Figure 1).

FIVs of different strains are found in both domestic and wild feline species, including cheetah, lion, puma, bobcat, leopard, and Pallas’ cat [8,9,10,11,12]. Phylogenetic analyses of FIV from many species of Felidae demonstrate that FIV lineages are species-specific, and also suggest that the FIV/host co-evolution existed over a long period [13,14]. In addition, cross-species transmission of FIV between different feline species has been observed [8,15,16,17,18]. However, phylogenetic evidence indicates that these FIV transmissions are exceedingly rare events between wild feline species, and that restriction factors of the host may act as a barrier and thus prevent the spread of FIV [8,9,19].

**Figure 2:**
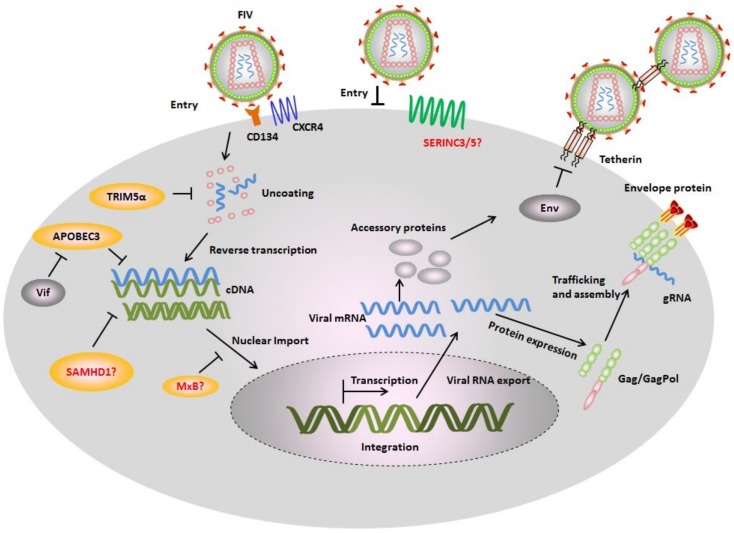
Feline restriction factors and FIV counteraction mechanisms. In the absence of viral antagonists, several cellular proteins called restriction factors inhibit different stages of viral replication cycle. Monkey tripartite motif-containing protein 5α (TRIM5α) interacts with FIV capsid and inhibits an early infection step. Felines expresses a truncated *TRIM5* gene that appears to have no antiretroviral activity, while the artificial fusion protein of feline TRIM5 with feline cyclophilin A (CYPA) displays potential inhibition against FIV. Feline apolipoprotein B mRNA-editing enzyme, catalytic polypeptide-like 3 (APOBEC3) induce hypermutations of FIV genomes by its cytidine deamination activity. It is still unknown whether feline expresses serine incorporator protein 3/5 (SERINC3/5) and whether they contain antiviral activity. Cats have mutation in the MxB genes resulting in a very short transcript not encoding a functional protein. Feline tetherin prevents FIV release from cell surface. The restriction factors are counteracted by FIV encoded proteins. FIV Vif interacts with feline APOBEC3s and induces their degradation by the proteasome pathway. FIV Env counteracts the restriction of tetherin.

Restriction factors are cell-intrinsic proteins that can potently suppress the replication of lentiviruses. In some cases, restriction factors are induced by interferons (IFN). Many restriction factors have been identified that can suppress the replication of human immunodeficiency virus-1 (HIV-1), simian immunodeficiency virus (SIV), and FIV. The best-studied examples are TRIM5α (tripartite motif-containing protein 5α), APOBEC3 (apolipoprotein B mRNA-editing enzyme, catalytic polypeptide-like 3), SAMHD1 (SAM and HD domain-containing protein 1), MxB (myxovirus resistance B), tetherin, and SERINC3/5 (serine incorporator protein 3/5) [20,21,22]. TRIM5α interacts with the lentiviral capsid and inhibits viral uncoating, induces an innate signaling cascade, and may be involved in autophagy [23,24,25,26,27,28,29]. APOBEC3s decrease viral reverse transcription and induce hypermutation of the viral cDNA via the enzymes’ cytidine deamination activity (see recent review [30]). SAMHD1 reduces the cellular dNTPs (deoxyribonucleoside triphosphates) level and inhibits viral reverse transcription (see recent review [31]). MxB prevents viral nuclear import and integration [32,33]. Tetherin prevents viral release from the cell surface [34]. SERINC3/5 can be packaged into viral particles and inhibit viral entry via an Env-dependent mechanism [21,22] (Figure 2).

Feliformia express a truncated *TRIM5* gene that appears to have no antiretroviral activity [35]. However, the fusion protein of feline TRIM5 with feline cyclophilin A (CYPA) displays potent inhibition against FIV and HIV-1 [36]. Similar to cat *TRIM5*, cat *MxB* has been shown to be mutated and inactivated [37,38]. One recent study indicates that feline SAMHD1 is expressed in a wide range of cat tissues, including skin and mucosal epithelium spermatogenic tissues, as well as FIV-susceptible cell lines [39]. No study has yet demonstrated whether feline SAMHD1 has the ability to restrict FIV or other retroviruses. However, stably expressed human SAMHD1 in human monocytic U937 cells inhibits the infection by FIV [40]. It is unknown whether felines express SERINC3/5 and whether they contain antiretroviral activity. Thus, the best-studied feline restriction factors are tetherin and APOBEC3. Feline tetherin can prevent the release of FIV and HIV-1, which is antagonized by the FIV envelope glycoprotein [41,42,43]. Feline APOBEC3 (A3) restricts the infectivity of Vif-deficient FIV (FIVΔVif), and FIV Vif counteracts the feline A3 antiviral activity [44,45,46].

## 2. FIV Vif and Feline APOBEC3

Deletion or mutation of *vif* in HIV-1 and HIV-2 causes significant defects in viral replication in many primary lymphoid and myeloid cell lines that are termed “non-permissive” cells [47,48,49,50,51,52]. Despite limited sequence similarity between FIV Vif and HIV Vif, FIV Vif is also important for FIV replication. One previous study indicated that FIV *vif* is required for productive infection of FIV in feline peripheral blood mononuclear cells and monocyte-derived macrophages [53]. Another study showed that challenging newborn kittens with FIVΔVif does not cause the production of anti-FIV antibodies, and also does not alter the CD4 T-cell count [54]. Moreover, Paul et al. reported that Vif mutations in FIV isolated from a non-domestic felid, *Otocolobus manul*, called FIV-Oma causes attenuated viral replication in feline CrFK cells that naturally express A3s [55].

An investigation of the HIV-1 Vif function first identified human APOBEC3G (apolipoprotein B mRNA editing enzyme, catalytic polypeptide-like G or A3G) as a restriction factor that inhibits HIV-1ΔVif and is suppressed by HIV-1 Vif [56]. A3s are either constitutively expressed or interferon-induced cellular enzymes that contain either one or two zinc-binding (Z) domains with the conserved motif HXE(X)_23–28_CXXC (where X is any residue) [57]. This Z domain includes three distinct phylogenetic clusters designated Z1, Z2, and Z3, respectively [57]. The A3 family of DNA cytidine deaminases is found in placental mammals with different clade-specific gene copies and arrangements, and A3 plays a vital role in innate immune defense against retroviruses (see reviews [30,58,59]). A3 proteins can be packaged into viral particles by interacting with viral Gag protein and RNA [60,61,62,63,64,65]. In the next round of infection, A3 inhibits viral replication by deamination of cytidines in the viral cDNA that is formed during the reverse transcription step by introducing G-to-A hypermutations in the coding strand [56,66,67,68,69]. The uracil-containing viral cDNAs are partially destroyed by cellular DNA degradation enzymes [70], and the double-stranded DNA harboring G-to-A hypermutations would code for viral proteins with premature stop codons or mutated proteins. In addition, some A3s inhibit viral replication by decreasing reverse transcription and integration via deaminase-independent mechanisms [71,72,73,74,75,76]. One recent study demonstrated that human A3G directly interacts with HIV-1 reverse transcriptase and inhibits its function [77].

**Figure 3:**
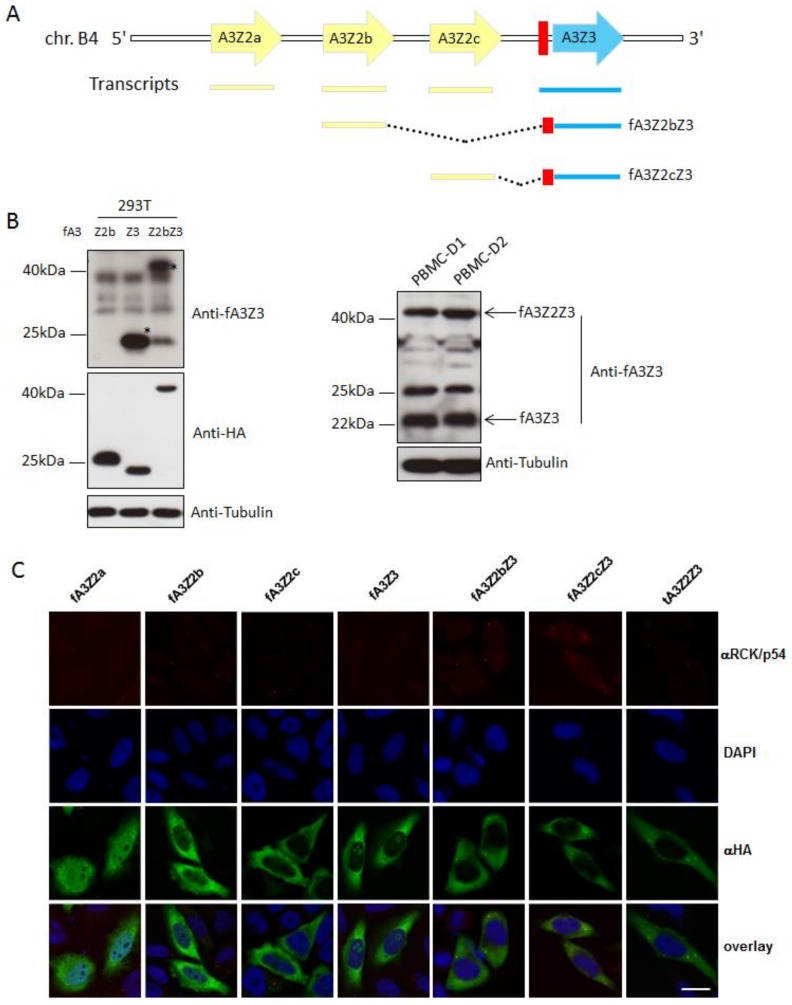
The expression and cellular localization of feline APOBEC3s. (**A**) Representation of *APOBEC3* (A3) genes in the genome of *Felis catus*. Coding regions of the A3 genes (*A3Z2a*, *A3Z2b*, *A3Z2c* and *A3Z3*) are shown as arrows. Red rectangle: exon 2 of *A3Z3* that is untranslated in the *A3Z3* mRNA, however translated (“linker domain”) in read-through transcripts A3Z2bZ3 and A3Z2cZ3; (**B**) 293T cells transfected with feline A3Z2b, A3Z3, and A3Z2bZ3 expression plasmids were harvested for immunoblotting by using polyclonal rabbit serum raised against fA3Z3 and anti-HA antibodies. Asterisks indicate the detection of feline A3Z3 and feline A3Z2bZ3 proteins. Cat PMBCs from two donors were stimulated with concanavalin A and then cultured with 100 U/mL IL-2. The Expression of cat A3Z3 and A3Z2Z3 proteins in stimulated peripheral blood mononuclear cells (PBMCs) were detected by immunoblots using anti-fA3Z3 antibody. Tubulin served as a loading control; (**C**) Hela cells were transfected with feline A3s expression plasmids with a HA tag. To detect A3 (green) immunofluorescence, staining was performed with an anti-HA antibody. Nuclei (blue) were visualized by 4′,6-diamidino-2-phenylindole (DAPI) staining. The cellular P-bodies were visualized by staining with anti-RCK/P54 antibody. Bar = 20 µm. f indicates feline, t indicates tiger.

Humans have several A3 genes coding for seven proteins A3A–A3D and A3F–A3H [57,59], while the domestic cat expresses four single-domain A3s (A3Z2a–A3Z2c and A3Z3), as well as double-domain A3Z2Z3 proteins by read-through transcription and mRNA alternative splicing [44,45] (Figure 3A). A previous study quantified the feline A3 mRNA level in CrFK cells, Mya-1 feline T cells, and cat peripheral blood mononuclear cells (PBMCs), indicating that feline *A3Z2* mRNA expression is highest, the expression of feline *A3Z3* mRNA is moderate, while the feline *A3Z2Z3* mRNA level is lower [78]. However, at the protein level, feline A3Z3 and A3Z2Z3 are likely expressed at a similar level in activated feline PBMCs (Figure 3B).

We also investigated the subcellular localizations of feline A3s and showed that the feline A3Z2a protein was distributed in both the cytoplasm and the nucleus, while A3Z2b and A3Z2c mainly localized in the cytoplasm (Figure 3C). Feline A3Z3 localized in the cytoplasm with additional distribution in the nucleoli (Figure 3C). Feline double-domain A3 proteins were distributed in the cytoplasm with protein accumulation in puncta (Figure 3C).

**Figure 4:**
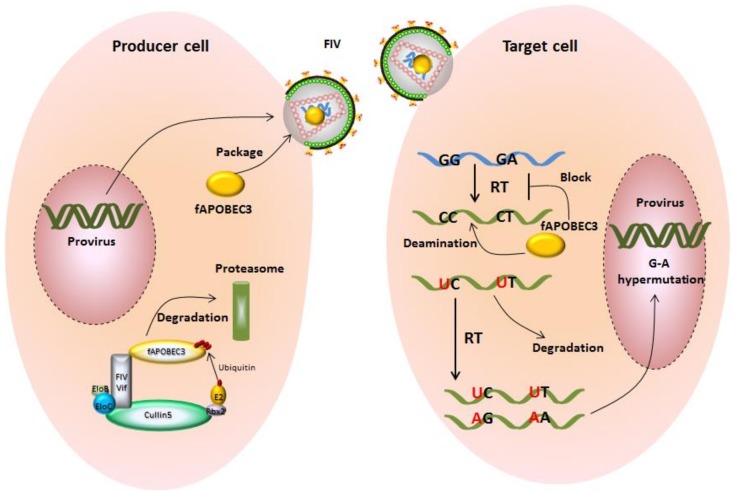
Feline A3s inhibit the replication of FIV and are counteracted by FIV Vif. In producer cells, A3s can be incorporated into virus particles and be delivered into target cell in the absence of Vif. During viral reverse transcription, virion-encapsidated A3s are able to catalyze the cytidine to uridine in viral cDNA. In the synthesis of next strand virus DNA, G-A hypermutations are produced on the coding strand.The uracil-containing viral cDNAs are partially degraded by cellular DNA repair enzymes, and the double-stranded DNAs harboring G-to-A hypermutations encode viral proteins with premature stop codons or mutated proteins. However, FIV Vif directly interacts with feline A3s and forms an A3-Vif-E3 ubiquitination complex, which induces A3 degradation by the proteasome pathway. In Vif expressing FIV infections, viral particels are produced that are mostly free of A3 proteins.

Similar to human A3s, feline A3s can reduce retroviral infectivity. Under experimental conditions, feline A3Z3 moderately suppresses FIVΔVif infection (around 5-fold), while feline A3Z2Z3 strongly inhibits FIVΔVif (around 20-fold) [45,79]. It remains unclear why feline A3Z2s have no antiviral activity against FIVΔVif. Both feline A3Z3 and A3Z2Z3 are able to introduce G-to-A hypermutations in Vif-deficient FIV genomes via their cytidine deaminase activity [44] (Figure 4), but it is unknown whether feline A3s can suppress FIVΔVif by deaminase-independent mechanisms. In addition to feline retroviruses, feline A3s also show antiviral activity against HIV-1, feline leukemia virus (FeLV), and feline foamy virus not expressing the Bet protein (FFVΔBet), as well as a rather weak inhibition of equine infectious anemia virus (EIAV) [44,45,80,81,82,83].

To antagonize the restriction of feline A3s, FIV Vif induces the degradation of feline A3s in the virus-producing cells and thereby prevents their packaging into viral particles [44,45]. In FIV-infected feline CrFK cells and human cells with transiently expressed FIV Vif, the Vif protein localizes in both the cytoplasm and the nucleus [79,84], but whether the nuclear localization of Vif is important for feline A3s antagonism and FIV replication is unknown. FIV Vif, similar to HIV-1 Vif, forms an E3 ubiquitin ligase complex that contains elongin B/C, cullin5 (CUL5), and ring box-1 to induce feline A3 degradation [85] (Figure 4). While CBF-β (core-binding factor subunit beta) is an essential cofactor for HIV-1 and SIV Vifs to form a stable E3 ligase complex [86,87], FIV Vif does not interact with CBF-β. In addition to FIV, Vifs of other non-primate lentiviruses—e.g., maedi-visna virus (MVV), caprine arthritis encephalitis virus (CAEV), and bovine immunodeficiency virus (BIV)—degrade A3s without CBF-β binding [88,89,90,91]. A recent study demonstrated that BIV Vif appears to operate independently of any cofactors, while MVV Vif hijacks cellular CYPA as a cofactor to reconstitute the E3 ligase complex [88]. It is unclear whether FIV Vif recruits any additional protein(s) to stabilize this E3 ligase complex.

**Figure 5:**
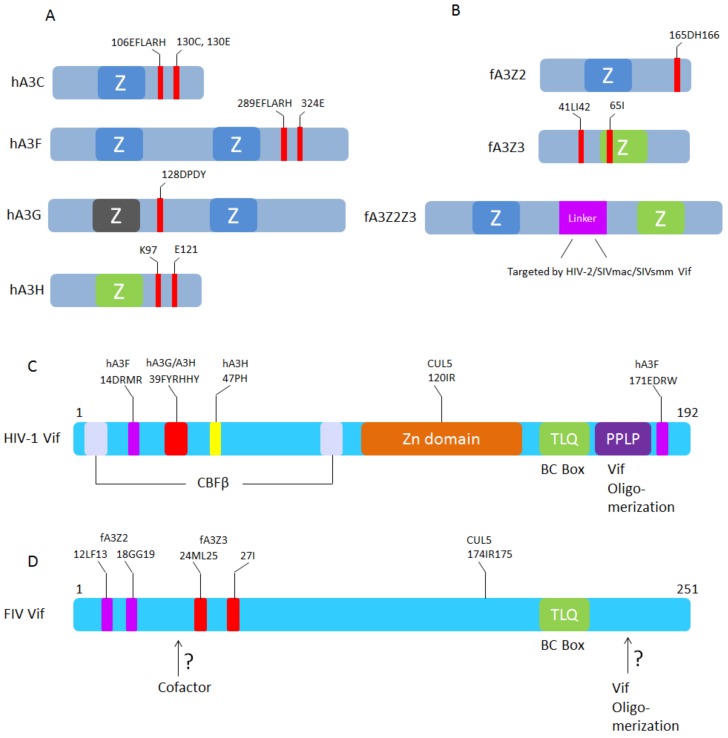
Schematic representation of A3-Vif interaction sites. (**A**) HIV-1 Vif binding sites in human A3C, A3F, A3G, and A3H [60,94,95,96,97]; (**B**) FIV, HIV-2, SIVmac, and SIVsmm Vifs binding sites in feline A3Z2, A3Z3, and A3Z2Z3, respectively [79]; (**C**) The human A3F, A3G, A3H, CUL5, CBF-β, and ElonginB/C binding sites in HIV-1 Vif (see review [98]); (**D**) The feline A3Z2, A3Z3, and CUL5 binding sites in FIV Vif [46]. FIV Vif domains that interact with additional cofactors and sites for oligomerization are not identified.

The FIV Vif-induced degradation of A3s is strictly limited to A3s of Felidae, and FIV Vif is unable to degrade A3s from non-felid species [45,83]. In contrast, SIV from rhesus macaque (SIVmac) Vif has a broad degradation activity for A3s. Interestingly, recent studies indicated that SIVmac, SIVsmm (SIV from sooty mangabey monkey), and HIV-2 Vifs are able to induce the degradation of feline A3Z2Z3 by targeting its unique linker domain [79,82,91] (Figure 5), which may hint at ancient and ongoing conflicts between lentiviral Vif and mammalian A3s [92]. The linker domain of feline double-domain A3 is unique to Feliformia and it is located in the 5’UTR of the felid *A3Z3* gene in exon 2, which is translated in read-through transcripts spanning the *A3Z2* and *A3Z3* genes in felines [45,79] (Figure 2). Deletion of this linker domain of feline A3Z2Z3 slightly reduces its antiviral activity against SIVΔVif and FIVΔVif [79]. It is unknown whether the recruitment of the linker as a coding sequence into feline A3Z2Z3 is related to the gain of other biological functions. Interestingly, one report indicated that the FIV protease is able to target the 230HSLH region of the feline linker domain and cleaves feline A3Z2Z3 in viral particles, which indicates that the protease has anti-feline A3 activity [93].

Several recent studies have characterized the molecular interactions of FIV Vif with feline A3s and FIV Vif with the E3 ligase complex. One study indicated that feline A3Z3 haplotype V (I65) has been selected for escape from ancestral FIV by its resistance to FIV Vif-induced degradation [99] and this A3 polymorphism correlated with susceptibility to FIV and FeLV infections [100]. Similar polymorphisms of human A3H (Z3 type A3) were also described and these may play a role in SIV from chimpanzee (SIVcpz) cross-species transmission, HIV-1 infection, and acquired immune deficiency syndrome (AIDS) progression [101,102,103,104,105,106,107,108,109,110,111,112,113]. In a follow-up study, residues 41LI42 of feline A3Z3 were identified as being essential for FIV Vif interaction and degradation, and a combination of A65I and L41A–I42A in feline A3Z3 completely blocks FIV Vif binding and degradation [79]. In feline A3Z2, the residues 165DH166 regulate whether FIV Vif can induce degradation, and only feline A3Z2Z3 that combines D165Y with H166N in the Z2-domain and A65I + L41A, I42A in the Z3-domain is resistant to FIV Vif-induced degradation. Structural homology modeling indicates that the FIV Vif binding regions in feline A3s show a unique location that is different from those described for HIV-1 Vif in human A3s [79] (Figure 5B).

FIV Vif selectively degrades feline A3Z2 and A3Z3 by using its N-terminal residues [46]. As the feline A3s show distinct Vif interactions, the FIV Vif protein evolved different amino acids that selectively target either feline A3Z2 or A3Z3. Residues 12LF13 and 18GG19 of FIV Vif specifically interact with feline A3Z2, while 24ML25, and 27I exclusively regulate the degradation of feline A3Z3 (Figure 5D). These essential sites in Vif are conserved in different FIV subtypes suggesting a biological significance of retaining feline A3s’ counteraction during FIV evolution [46].

To characterize the molecular interaction in the FIV Vif-E3 ligase complex, Wang et al. identified the BC box (TLQ) of FIV Vif, which interacts with elongin B/C [85]. The CUL5 interaction site was recently characterized to be a hydrophobic region (174IR175) in FIV Vif [114]. A novel homology structure model of the FIV Vif/CUL5 complex indicates that the CUL5 interaction surface in the diverse HIV-1 and FIV Vifs is evolutionarily conserved [114].

## 3. Feline Foamy Virus and Feline Leukemia Virus

In addition to FIVΔVif, feline APOBEC3s also inhibit the replication of FFV [80]. Feline A3Z2s strongly restrict the feline foamy virus ΔBet (FFVΔBet), but feline A3Z3 and A3Z2Z3 only slightly decrease FFVΔBet infectivity [44]. Gag binding efficiency of feline A3s may directly correlate with the level of restriction of Bet-deficient FFV [115].

Similar to the FIV Vif protein, FFV Bet binds all feline A3s independent of whether they restrict FFV or not [80,115]. In contrast to Vif, FFV Bet does not induce degradation of feline A3s, but the binding of Bet to feline A3s prevents their viral incorporation [80]. The Bel2 domain of FFV Bet is essential for the inactivation of feline A3s, whereas the Bel1 domain increases Bet stability and can be exchanged by related sequences [116], suggesting that FV Bet prevents A3-mediated restriction of viral replication by creating strong complexes with these proteins [116].

The Bet of prototype foamy virus (PFV), a chimpanzee virus that was isolated from human cells [117], also counteracts the restriction by human A3s [118,119,120]. PFV Bet antagonizes human A3C by forming a complex with A3C without inducing its degradation, and this counteraction is species-specific [120]. PFV Bet also impairs the dimerization and cytosolic solubility of human APOBEC3G [119]. Taken together, these data indicate that foamy viruses also developed an A3-counteracting protein that, unlike lentiviruses, uses a degradation-independent mechanism.

Feline leukemia viruses (FeLV) are gammaretroviruses that infect domestic cats. The infection of non-domestic felids by FeLV is rare and the viruses display self-limiting replication [121]. Investigations into the interaction of FeLV with feline or non-feline A3s are limited [44,122].

## 4. FIV Cross-Species Transmission

There is a worldwide distribution of FIV infection in domestic cats (FIVfca) with several FIV subtypes recognized in different countries [3]. The main pathway of FIV transmission in cats is via bite wounds, although vertical transmission of FIV has also been observed [123,124,125]. FIVfca transmission from domestic cats to wild cats is not well described, but seems to be plausible where domestic cats are eaten by other Felidae. One previous study reported the isolation of FIVfca from Tsushima Leopard cats (*Prionailurus bengalensis*) [15]. Vif from FIVfca can induce the degradation of A3s from both the domestic cat and several big cats [45], suggesting the possibility of transmission of FIV from domestic cat to wild big cats.

In addition to the domestic cat, FIV also infects big wild cats, such as lion (FIVple), puma (FIVpco), and bobcat (FIVlru) [12]. Interestingly, FIVple and FIVpco are able to replicate in a domestic cat T-lymphoma cell line [126]. The FIV that infects puma and bobcat is named as FIVpco and FIVlru, respectively, while some studies describe this virus as puma lentivirus (PLV) [8,9,13,16,18,127]. PLV includes subtypes A and B (PLVA and PLVB). PLVB infects puma throughout North and South America. PLVA infects puma and bobcats in southern California and Florida [18]. PLVA of puma is considered to be the result of an ancestral transmission event of PLVA from bobcat, and the cross-species circulation of PLVA in puma and bobcat is ongoing [16,128]. Lee et al. recently revealed that the *vif* gene from PLVA of puma was under selection, indicating an intrinsic restriction pressure of puma A3s [16]. However, there are no direct studies showing the restriction by puma A3s against PLVA, and we only have information that puma A3s display antiviral activity against FIV from domestic cat [45,129]. Thus, further investigations are required to test the restriction of puma A3s for its host lentiviruses, and also to test the interaction between PLVA Vif and puma A3s. Interestingly, one recent study showed that PLVB Vif is incapable of counteracting puma A3Z3 [129]. However, the PLVB Vif that was used in this study was derived from strain PLV-1695, which resulted from several passages in domestic cat [130], and it would be interesting to evaluate the counteraction activity of the PLVB Vif currently circulating in wild puma for puma A3s.

The A3s restriction factors are likely important to prevent lentiviral transmission between distantly related mammals. The impact of A3s on lentiviral cross-species transmission in more closely related species of a family clade such as primates or felids is only partially understood [45,79,99,100,131]. While some laboratory data on the activity spectrum of different FIV Vifs indicate the potential to counteract A3s of non-autologous Felidae host species, observations in captive or wild animals suggest that FIV cross-species transmission is rare.
